# Leachate Analysis of Heavy Metals in Cigarette Butts and Bricks Incorporated with Cigarette Butts

**DOI:** 10.3390/ma13122843

**Published:** 2020-06-25

**Authors:** Halenur Kurmus, Abbas Mohajerani

**Affiliations:** School of Engineering, RMIT University, Melbourne 3000, Australia

**Keywords:** leachate analysis, cigarette butts, recycling, fired clay bricks, waste management

## Abstract

Billions of cigarette butts (CBs) are discarded as litter in the environment every year worldwide. As CBs have poor biodegradability, it can take several years for them to break down while leaching toxic chemicals and heavy metals. Mohajerani et al. (2016), based on long-term research, developed a method for the recycling of CBs in fired clay bricks with promising results. This paper presents and discusses the leaching behavior of potentially hazardous metals from used, unused, and shredded used CBs, and unfired and fired clay bricks incorporating CBs. The leachate analysis was conducted according to the Australian Bottle Leaching Procedure (ABLP) for pH values 2.9, 5.0, and 9.2. The aim was to quantify the amount of heavy metals leached, determine the relationship between the metal concentration leachate, pH of the solution and condition of the sample, and examine the effect of firing on the leaching capability of bricks. The leachate results were then compared to the concentration limits for heavy metals set by the United States Environmental Protection Authority (USEPA) national primary drinking water and the Environmental Protection Authority (EPA) solid industrial waste hazard categorization thresholds to assess the suitability of fired clay bricks incorporating CBs. Metals Cu, Zn, Mn, Al, Fe, Ti, and Ba demonstrated the highest leachate concentrations for pH 2.9 and pH 5.0 for used CBs. This suggests that used CBs are more prone to leaching heavy metals in areas with highly acidic rain compared to the natural range of precipitation. The leaching behavior of fired bricks incorporating CBs was considerably lower than that for the unfired bricks due to the immobilization of heavy metals during the firing process. However, the leaching of Cr and Ni was almost completely impeded after the firing of the bricks, and more than 50% of all the tested heavy metals were hindered.

## 1. Introduction

The littering of cigarette butts (CBs) has become a critical environmental issue around the world. In Australia, 20 billion filtered cigarettes are consumed annually, and it is predicted that over 7 billion are thrown into the environment [1]. In 2016, 5.7 trillion cigarettes were consumed worldwide, and it is estimated that by 2025, cigarette production will increase by 50% [2].

Over 97% of cigarettes have cellulose acetate filters [2], which are designed to partially preserve particulate smoke components. The major toxic agents include nicotine, polycyclic aromatic hydrocarbons (PAHs), compounds specific to Solanaceae, nicotiana alkaloids, and catechols [3,4]. Cellulose acetate filters have poor biodegradability, and, under normal environmental conditions, they can take up to 18 months to decompose; thus, becoming a source of toxic waste, polluting waterways and groundwater, and harming aquatic life and animals [5]. Several companies in Australia have developed CB disposal systems consisting of receptacles, and the service includes the removal and final disposal of the CB waste. The common methods implemented for the disposal of collected CBs include landfilling and incineration. However, neither technique is economically viable or environmentally friendly [6]. Therefore, it is possible for companies to directly deliver CB waste to brick-manufacturing facilities to be used for recycling. 

The prevalent heavy metals found in CBs that prompt a likely risk to human health, aquatic organisms, and the environment, are Na, As, Mg, Sc, Pb, La, Bi, Cr, Mn, Cs, Co, Ni, Cu, K, Ag, Zn, Cd, Fe, Hg, Al, Sb, Au, Se, and Te [7,8]. To investigate the transfer of toxic metals in the tobacco to the smoke component, several studies have been conducted [9,10,11]. Benson et al. [12] found that the concentrations of Cu, Mn, Pb, and Zn are relatively higher in the filler tobacco compared to the concentration in the smoked cigarette filter, except for Cd. Dobaradaran et al. [13] found that Hg and Pb may be potential sources of prolonged contamination for aquatic organisms in coastal areas based on their response to heavy metals [13]. From a general point of view, Ar and Cr are causally associated with cancer in humans, while Ni and Cd are probably carcinogenic to humans [10]. Heavy smokers accumulate more than twice the body burden of Cd compared to non-smokers. The inhaled heavy metals may prompt disorders of mineral metabolism, such as osteoporosis [7]. 

A promising approach to a growing pollution calamity may be through the recycling of CBs in fired clay bricks. Mohajerani et al. [14] carried out a comprehensive study on recycling 0%, 2.5%, 5% 7.5%, and 10% CBs in fired clay bricks with promising results. The tests carried out include density, compressive strength, water absorption, initial rate of absorption, shrinkage, modulus of rupture, thermal conductivity, and energy savings. A decrease in compressive strength was observed from 25.65 MPa to 12.57 MPa for bricks incorporating 0% and 2.5% CBs, while water absorption decreased from 18% to 5% for bricks incorporating 7.5% and 0% CBs. The study found an increase in CB content resulted in less dense clay bricks with greater porosity and consequently improved thermal performance and energy savings. A comprehensive study was completed for 1% CBs in fired clay bricks for the confirmation of the results. The compressive strength for controlled brick decreased from 25.65 MPa to 19.52 MPa for bricks incorporating 1% CBs, and water absorption increased from 5% to 7.4% with the addition of 1% CBs. The paper proposed that by incorporating 1% CBs in 2.5% of the world’s brick production, all CBs can sustainably be recycled [15,16,17].

Several other investigations have been conducted to recycle waste in the production of fired clay bricks. Such waste includes waste marble powder [18], recycled paper processing residue [19], sugarcane bagasse ash [20], olive pomace [21], bio-briquette ash [22], expanded vermiculite [23], and olive mill solid residue [24]. However, placing toxic wastes in construction materials poses serious exposure risks to the environment and human health. Consequently, it is essential to conduct comprehensive environmental valuations of innovative building materials incorporating waste prior to their use as commercial products. 

The main environmental issues raised from the use and disposal of construction materials incorporating various hazardous wastes are the possible leachate of toxic chemicals and heavy metals to the surrounding environment. Therefore, it is essential to conduct a comprehensive leachate analysis to classify and quantify those harmful constituents. Currently, there are more than 50 leaching tests available for various sample conditions, pH values, and solid-to-liquid ratios to mimic a range of possible environmental conditions. A leachate test determines the liquid that may liquify or dissolve toxic chemicals or heavy metals and potentially enter the environment [25]. The methods vary in terms of the mass and particle size of the sample, the type and volume of the leachate fluid, the leachate delivery method, and the leachate contact time. The main leachate methods have been summarized in Table 1, including their applications and limitations.

By exploring the various leachate testing methods and comparing their applications and limitations, it is apparent that each method is limited to a specific material or environment. For construction materials that are exposed to harsh weather conditions, in particular, the pH of the solution is very critical. Therefore the Australian Bottle Leaching Procedure (ABLP) [26] or Method 1313 [29] testing methods are recommended as both are pH dependence tests and are suitable for fired clay bricks. The leachates from CB bricks represent heavy metals, which can possibly leach into the surrounding environment, and pose significant concerns for the quality of aquatic and urban life. The Leaching Environmental Assessment Framework (LEAF) is the most comprehensive method due to its extensive scope and comprises of Methods 1313, 1314, 1315, and 1316 [29,30,31,32]. However, the four methods in LEAF are recommended to be used in conjunction when conducting a leachate analysis and can be time consuming and unnecessary. 

Therefore, in reviewing the various leaching test methods, the ABLP [26] was determined to be the most suitable leaching procedure for this particular study based on the sample size, pH values, disposal-to-land scenarios, and condition of the sample in comparison to the USEPA methods. Previous studies concerning the leachate of bricks incorporating CBs have found the concentrations of the metals to be in trace amounts and less than the regulatory thresholds [14]. However, the framework and methodologies followed did not account for the leaching behavior of bricks incorporating CBs over the scope of potential environmental situations expected in various disposal scenarios. In addition, the procedure followed was based on the Australian Standards edition published in 1997.

This paper is the continuation of an ongoing study on the incorporation of CBs in fired clay bricks. The objective of this study was, therefore, to conduct two comprehensive leachate analysis investigations. In the first study, the leachate characteristics of used, unused, and shredded used CBs for various heavy metals were assessed. The aim was to quantify the amount of heavy metals leached from littered CBs and determine the correlation between the metal concentration leached and the pH of the solution. The second part of the study involved evaluating the leachate characteristics of unfired and fired clay bricks incorporating 0%, 0.5%, 1%, 1.5%, and 2% CB content by mass. The leachate concentrations of various heavy metals were examined for unfired and fired bricks and compared to study the effect of firing on the immobilization of heavy metals of fired clay bricks. The leachate tests were performed in accordance to the ABLP method [26] for three pH values; 2.9, 5.0, and 9.2, to investigate how the pH affects the leaching behavior of the metals studied over a wide range of possible conditions. To evaluate the suitability of the fired clay bricks incorporating CBs, the leachate results were compared to the concentration limits for heavy metals set by the solid industrial waste hazard categorization published by the Environmental Protection Authority (EPA) Victoria Industrial Waste Resource Guidelines [34] and the United States Environmental Protection Authority (USEPA) national primary drinking water thresholds [35]. 

## 2. Materials and Methods

### 2.1. Materials

Sandy silty clay (MC) and CBs were selected as the raw materials. The CBs of varying brands and sizes were supplied by Buttout Australia Pty Ltd. (Melbourne, Australia), while the soil was provided by PHG Bricks (Victoria, Australia). On arrival, the CBs were oven-dried at 105 °C (24 h) and disinfected. The oven-dried CBs were then placed in airtight plastic bags ready for use for the preparation of bricks samples. Particle size distribution (PSD) test was conducted on the brick soil using sieve analysis [36]. The brick soil was characterized by determining the mineralogical composition through an X-ray diffraction (XRD) analyzer (D8 Endeavor, Bruker, Billerica, MA, USA) and the chemical composition through X-ray fluorescence (XRF) (S4 Pioneer, Bruker, Billerica, MA, USA).

### 2.2. Preparation of Brick Samples

**Figure 1:**
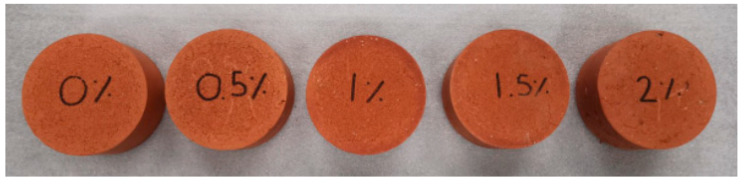
Some of the prepared brick samples with different percentages of cigarette butts (CBs).

Five different mixtures were prepared and used in this experiment: clay bricks with 0% CBs by mass and bricks made of clay and 0.5%, 1%, 1.5%, and 2% CBs by mass. The mechanical and physical properties of the prepared brick samples were assessed and prepared to be used for the leachate analysis test. Two batches of specimens were prepared and mixed in a Hobart mechanical mixer for 25 min with a water content of 15.5% and then compacted in cylindrical molds of 50 mm × 100 mm. The clay brick specimens were oven-dried at 105 °C. The oven-dried bricks were named as ‘unfired bricks.’ The second batch of unfired specimens was then fired at a temperature of 1050 °C for 3 h. The fired brick was named as ‘fired bricks.’ The fired brick samples were tested for the initial rate of absorption (IRA), compressive strength, shrinkage, water absorption, efflorescence, microstructural analysis, and density according to the Australian Standards [37] (Figure 1). All the results presented are the average of three replicates. 

### 2.3. Sample Preparation and Leachate Analysis

**Figure 2:**
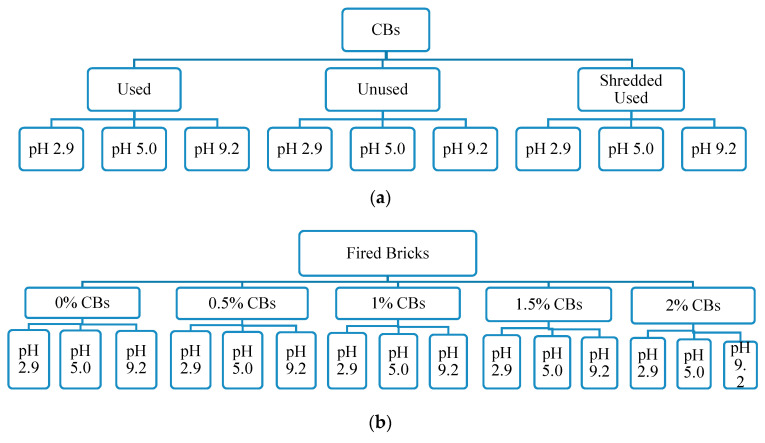

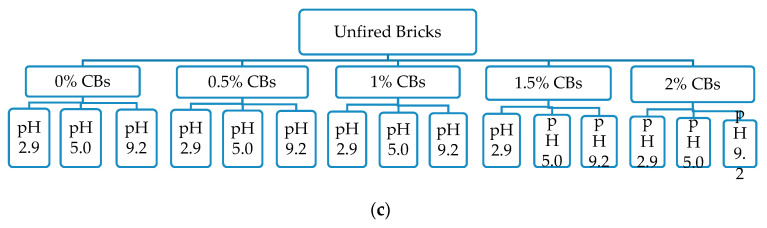
Leaching procedure for leachates derived from (**a**) used, unused, and shredded used CBs and (**b**) fired clay bricks and (**c**) unfired clay bricks incorporating 0%, 0.5%, 1%, 1.5%, and 2% CBs by mass.

**Figure 3:**
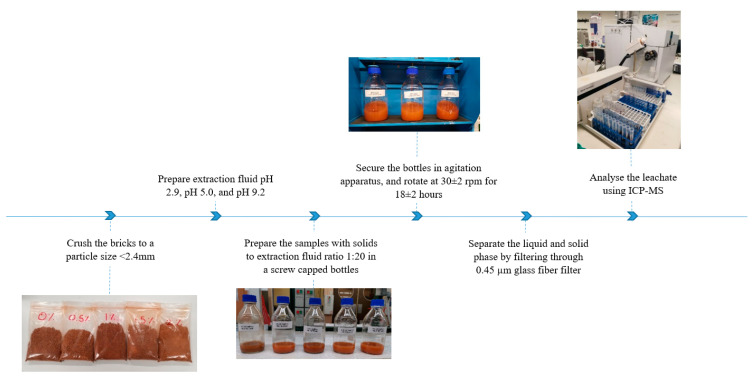
Flow chart of the leachate analysis procedure followed in this study.

To evaluate the relationship between the metal concentration leached and the pH of the leaching solution, and to simulate various environmental conditions, three pH values were selected, 2.9, 5.0, and 9.2 (Figure 2). The metals selected for both investigations were based on their toxicity levels to the local and aquatic organisms, their presence in CBs, and their ability to be analyzed easily by inductively coupled plasma mass spectrometry (ICP-MS). The leaching of heavy metals from the prepared brick samples was determined based on the ABLP [26]. Heavy metals, including cadmium (Cd), iron (Fe), aluminum (Al), lead (Pb), arsenic (As), mercury (Hg), barium (Ba), nickel (Ni), copper (Cu), titanium (Ti), chromium (Cr), and zinc (Zn), were analyzed for used, unused, and shredded used CBs. While chromium (Cr), copper (Cu), silver (Ag), barium (Ba), mercury (Hg), selenium (Se), nickel (Ni), cadmium (Cd), arsenic (As), lead (Pb), and zinc (Zn) were examined for unfired and fired clay bricks incorporating CBs. In the ABLP method as presented in Figure 3, the brick samples were crushed with a hammer and sieved using a 2.4 mm sieve.

The fluid extract of pH 2.9 was prepared by adding glacial acetic acid (5.7 mL) to 900 mL of water, which was later diluted to 1 L with water. pH 5.0 was prepared by adding glacial acetic acid (5.7 mL) to approximately 900 mL of water to which sodium hydroxide solution (64.3 mL of 1 mol/L) was added; the solution was then diluted to 1 L with water and mixed well. pH 9.2 was achieved by dissolving sodium tetraborate decahydrate (borax) (38.2 g) in approximately 900 mL of water and then diluting this to 1 L with water. The pH of this fluid was to be between 9.1 and 9.3, and where the pH was not between these levels, the fluid was discarded.

Samples were prepared based on a 1:20 ratio in screw cap bottles. The mass of the crushed fired clay brick samples used in this study was 10 g, while the volume of the extraction fluid was 200 mL. The bottles were then placed in an agitator and rotated at 30 ± 2 rpm for 18 ± 2 h. The fluid extract was attained by filtering the samples through a 0.45-microfilter to separate it from the solid phase. The ICP-MS was utilized to analyze the extracted samples for heavy metals. The results exhibited in this study are the average of three replicates.

## 3. Results and Discussion

### 3.1. Characterization of Raw Materials

**Figure 4:**
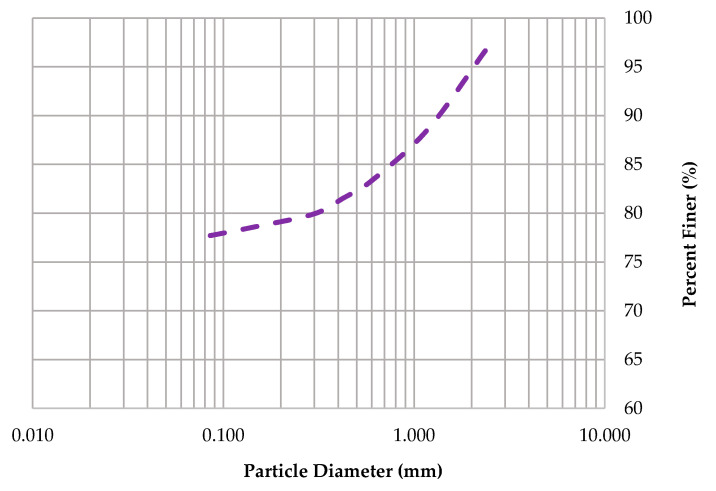
Particle size distribution (PSD) curve of brick soil used in this study.

**Figure 5:**
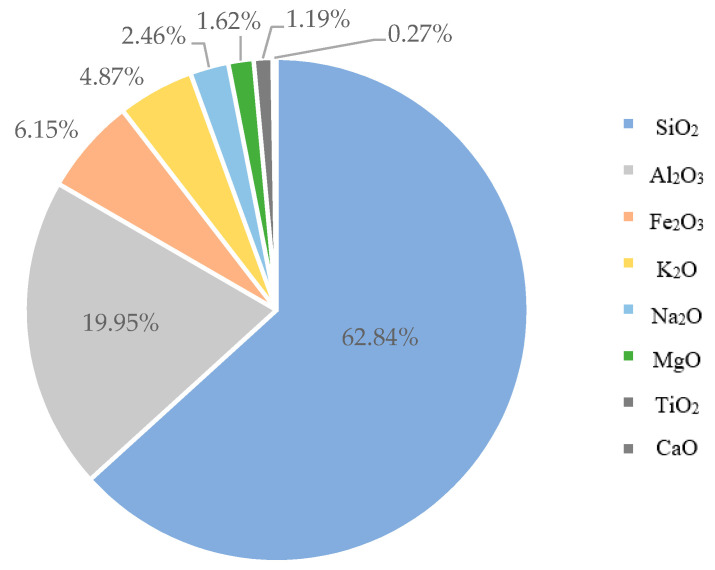
Brick soil chemical properties.

**Figure 6:**
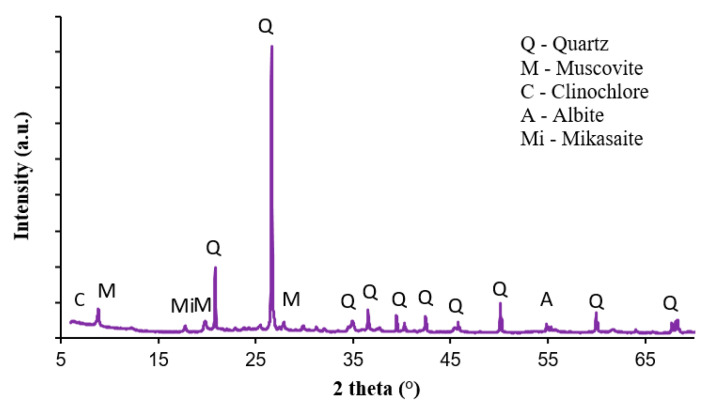
X-ray diffraction (XRD) patterns of brick soil.

The brick soil used in this study was sandy silty clay (MC) according to the unified soil classifications [36]. The results of the PSD are shown in Figure 4, which was carried out using sieve size analysis [36]. For the brick soil, the plastic limit was found to be 25% and 34% for the liquid limit. The XRF results of the chemical composition of the brick soil are given in Figure 5, while the XRD results of the mineralogical composition of the brick soil can be seen in Figure 6. It was observed that the main crystalline phase on a <75 µm soil sample of the brick soil was Quartz (SiO_2_).

### 3.2. Properties of Fired Clay Bricks

The properties of 0%, 0.5%, 1%, 1.5%, and 2% CB content by mass bricks are shown in Table 2 [38,39]. The compressive strength of a brick is a crucial quality for construction materials. In this study, the results indicate that the strength of the clay brick was significantly dependent on the quantity of CBs added. A decrease in compressive strength was found with the decrease in density and the increase in CB content. The results reveal that the compressive strength was in the range of 27.2–48.64 MPa, with a decrease in CB content from 2% to 0% by mass. Although there is a substantial decrease in strength with the increase in CB content, the values are considerably above the standard minimum compressive strength of 17.2 MPa for Grade MW (moderate weathering) bricks [40]. The density of the fired bricks was in the range of 1969–2114 kg m^−3^, with a decrease in CB content from 2% to 0% by mass. Light-weight bricks have several benefits in construction and production, including the reduction in transportation and labor costs and improving the thermal insulation properties of bricks.

The loss of water in the brick between the clay particles, which results in the proximate packing of those particles, is called firing shrinkage. The results show that the firing diametric and height shrinkage values decrease with the increase in CB content. It was found that the diametric shrinkage values were between 3.1% and 4.64%, with the decrease in CB content in the bricks from 2% to 0% by mass, which is below the maximum standard shrinkage value of 8% [41]. 

Water absorption determines the durability of a brick. When a high content of water can infiltrate a brick, the durability decreases, which can give rise to frost damage, salt crystallization, and change in volume, which can eventually result in structural damage to the building, therefore, a brick must be adequately dense to avoid the encroachment of water [15,42]. According to Table 2, water absorption and IRA increase with the increase in CB content. This is due to the decrease in density and the creation of pores from the CBs that were burnt off during the firing process. Bricks with a CB content of 0% to 2% by mass presented water absorption values between 9% to 13.1%. The water absorption for standard fired clay bricks should range between 5% to 20% [43]. The IRA values ranged between 0.44 to 0.84 kg m^−2^ min^−1^ for bricks incorporating between 0% to 2% CBs by mass. The values presented for IRA fall within the acceptable range of 0.2 to 5 kg m^−2^ min^−1^ [43]. Nevertheless, the water absorption and IRA results comply with the typical requirement range.

### 3.3. Leachate Analysis of Cigarette Butt (CB) Samples

Cigarettes contain a distinctive quantity of trace elements known to have toxicological effects on living organisms, and the smoking process drastically increases the concentrations of those elements in the cellulose acetate filter [10]. To identify and quantify the metals leached from discarded CBs and determine the relationship between the metal concentration leached, the pH of the solution and the condition of the sample, a comprehensive leachate analysis was conducted based on the ABLP [26]. 

To simulate natural conditions, the leaching procedures used in this study were designed as closely as possible to an open environment where CBs can be found. To investigate how the pH affects the leaching behavior of metals from CBs, three pH values were selected: 2.9, 5.0, and 9.2. The selected metals for this study include Ni, Zn, As, Hg, Al, Cu, Ba, Cr, Fe, Ti, Pb, and Cd and are based on their high degree of toxicity and presence in CBs [7,8,9,10,11].

**Figure 7:**
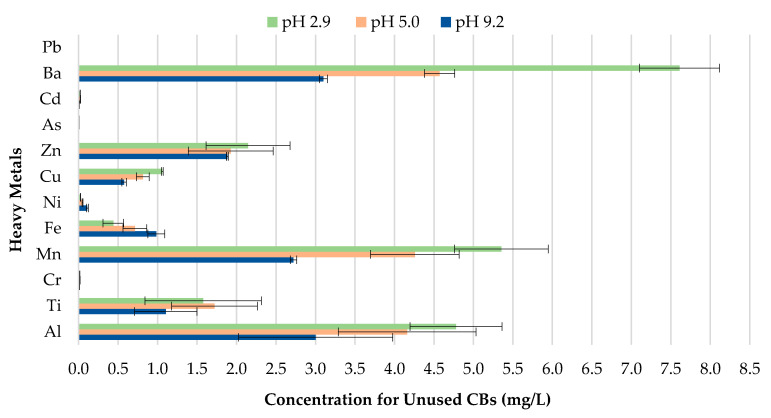
Leachate concentrations of unused CBs.

As illustrated in Figure 7, the findings suggest that differences in pH over a wide range of precipitation have an effect on the behavior of the metal concentrations leaching from unused CBs. A direct relationship between the concentrations of Cu, Zn, Mn, Al, and Ba leached, and the corresponding pH value was observed. Cu, Zn, Mn, Al, and Ba demonstrated the highest leachate concentration for pH 2.9, followed by pH 5.0; while pH 9.2 exhibited the lowest metal leachate concentration. The concentration level for Ba was 7.610, 4.572, and 3.1 mg/L for pH 2.9, 5.0, and 9.2. This implies that unused CBs are more likely to leach heavy metals in areas with highly acidic rain compared to the natural range of precipitation. This is due to the enhancement of metal mobilization in acid rain. The hydrogen (H^+^) ion in the acidic rain disturbs the cations (positively charged ion) from their connection sites, and this results in the decrement of cation exchange capacity and increase in the concentration of cations in CBs, therefore allowing the cations to be leached from the waste [44]. Likewise, the toxic ions (toxic metals) usually bound to the negatively charged surface and are displaced by H^+^ ion, too [45].

**Figure 8:**
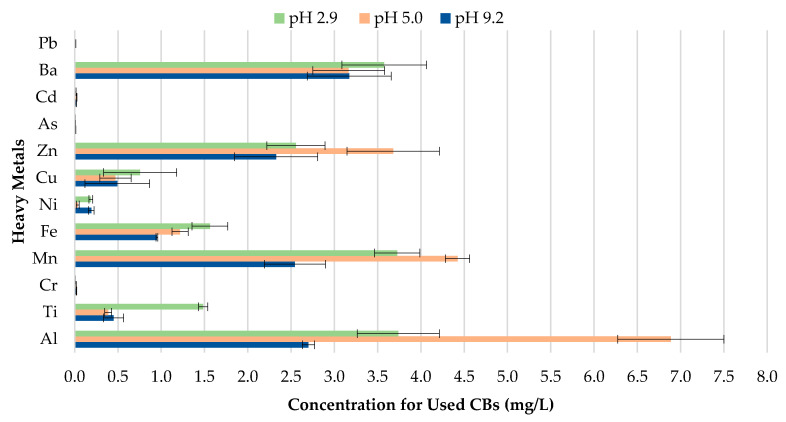
Leachate concentrations of used CBs.

The concentration of metals leached for used CBs for pH values 2.9, 5.0, and 9.2 can be seen in Figure 8. The results indicate that changes to the pH of precipitation will likely enhance or reduce the magnitude of metal contamination from used CBs. The metal concentration leachate for Zn, Mn, and Al for pH 5.0 was found to be much more significant compared to pH 2.9 and 9.2. In comparison, Ba, Cu, Ti and Fe depicted greater metal contamination for pH 2.9. Similarly to unused CBs, used CBs are more prone to leaching in highly acidic areas due to the enhancement of metal mobilization in acidic water. 

**Figure 9:**
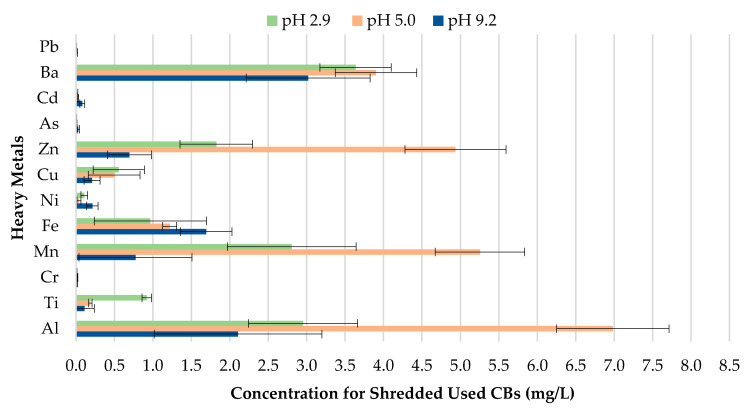
Leachate concentrations of shredded used CBs.

A direct relationship between the metal concentration leached and pH value was found for shredded used CBs. The concentration of the metals leached for pH values, 2.9, 5.0, and 9.2, can be seen in Figure 9. pH 5.0 resulted in the highest magnitude of metal contamination for Ba, Zn, Mn, and Al, followed by pH 2.9, while pH 9.2 demonstrated the least possible metal contamination. The relationship between metal leachate and pH value supports the results attained for both unused and used CBs. 

**Figure 10:**
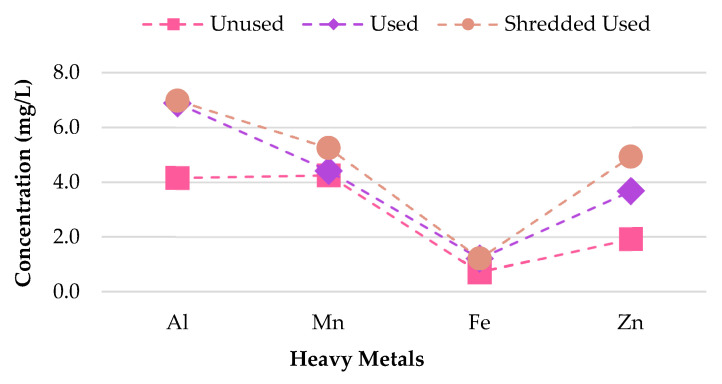
Al, Mn, Fe, and Zn leachate concentrations for pH 5.0.

To determine whether the smoking process influences the quantity of heavy metals found within CBs, the metal leachate concentrations for pH 5.0 for unused, used, and shredded used CB samples was analyzed. A linear relationship between the concentration of metals leached and the condition of the sample was found. Cr, Cd, Zn, Mn, Al and Fe contamination was greater for shredded used CBs compared to used and unused CBs (Figure 10). For shredded used CBs, 6.982 mg/L leachate was recorded for Al, compared to 6.887 mg/L leachate for used CBs and 4.161 mg/L for unused CBs. Generally, Al is one of the most abundant elements found in CBs due to its presence in tobacco. Al toxicity can have tissue-damaging effects, alteration in calcium metabolism in several organ systems, and Alzheimer’s disease [46]. The results suggest that used and shredded used CBs are more prone to leaching heavy metals Cr, Cd, Zn, Mn, Al and Fe compared to unused CBs. One explanation for this observation is that an enhanced level of Cr, Cd, Zn, Mn, Al and Fe is absorbed and retained by the filter during combustion. The remnant tobacco, which comprised of burnt and unburnt tobacco, effectively exacerbated the toxicity [47]. Other metals, including Pb and As are of insignificant concern for all sample conditions as their leachate concentrations are below 0.08 mg/L.

In contrast, the leaching of Cu, Ba, and Ni at pH 5.0 demonstrated a distinctive trend. Unused CBs exhibited the highest leachate concentration for all three metals, followed by shredded CBs, while used CBs displayed the least metal contamination. One explanation for this observation is that majority of the weight of Cu, Ba, and Ni pass through the vapor phase, contrary to being trapped in the filter.

### 3.4. Australian Bottle Leaching Procedure (ABLP) Leachate Analysis of Fired Clay Bricks

**Figure 11:**
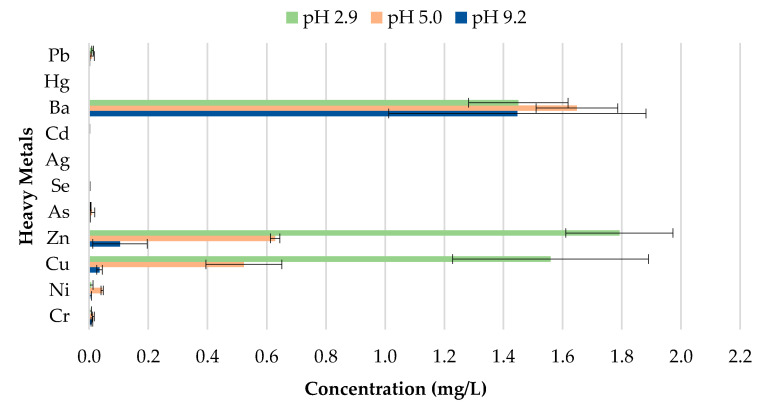
Leachate concentrations for crushed bricks with 0% CB content.

**Figure 12:**
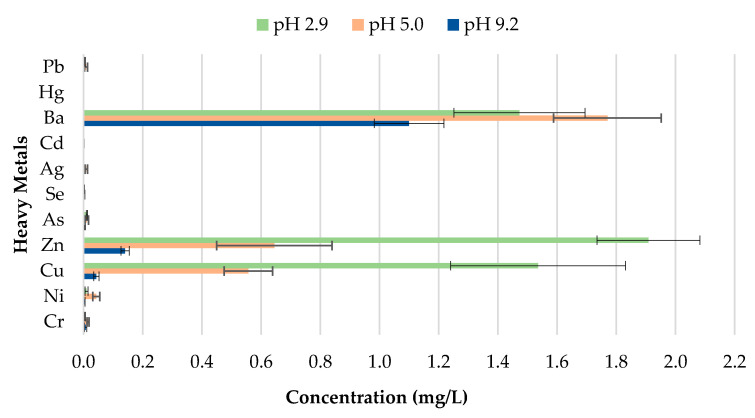
Leachate concentrations for crushed bricks with 0.5% CB content.

**Figure 13:**
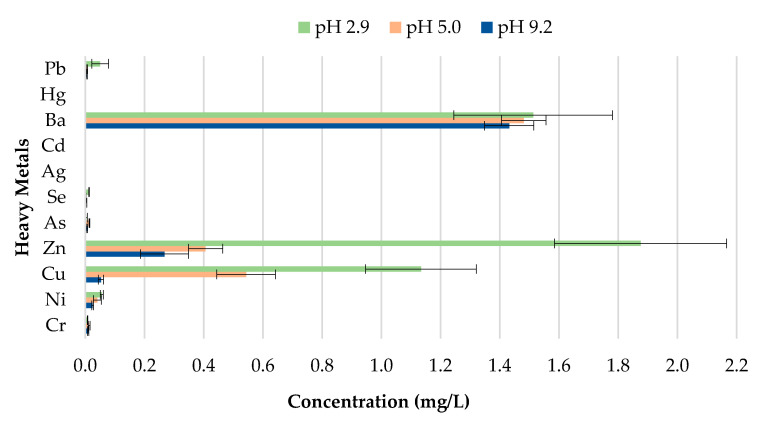
Leachate concentrations for crushed bricks with 1% CB content.

**Figure 14:**
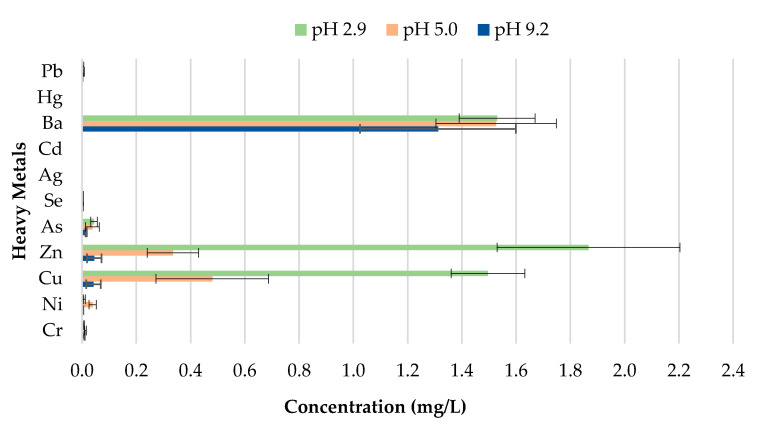
Leachate concentrations for crushed bricks with 1.5% CB content.

**Figure 15:**
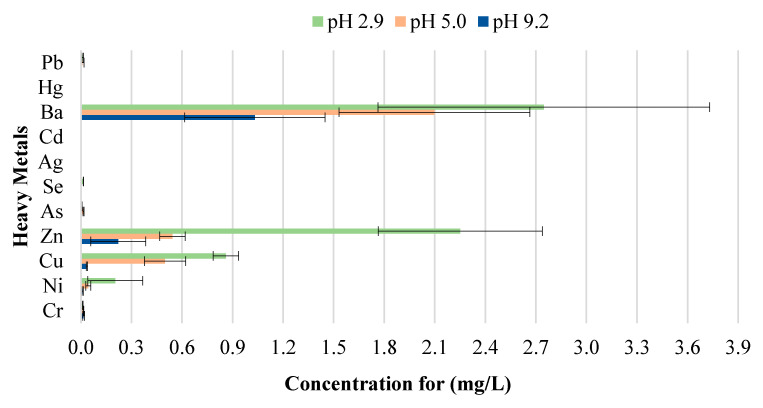
Leachate concentrations for crushed bricks with 2% CB content.

The toxicity of CBs is a concern when incorporated in construction materials like fired clay bricks. Therefore, it is essential to conduct extensive environmental valuations of innovative building materials modified with waste materials prior to being used as a commercial product. The transfer of heavy metals from bricks incorporating CBs to the environment is determined through a leachate analysis using the ABLP [26] and compared with the threshold regulatory limits for heavy metals set by the USEPA [35] and Industrial Waste Resource Guidelines [34]. Table 3 and Figure 11, Figure 12, Figure 13, Figure 14 and Figure 15 summarize the ICP-MS results for heavy metals using the ABLP method for pH 2.9, pH 5.0, and pH 9.2. All data are means of three values relative to standard deviation. The standard deviation is relatively small for most of the results, confirming the efficiency of the method.

The findings of this study indicate that differences in pH have an effect on the quantity of metal concentration leached for fired clay bricks incorporating CBs. The results imply that changes to the pH will likely increase or decrease the magnitude of metal contamination from fired clay bricks. In this study, an increasing trend in the metal concentration leachate was observed for fired clay bricks with the increase in CB content from 0% to 2% by mass. This is due to the increase in CB content, as discussed above; CBs are more prone to leaching heavy metals in areas with a pH of 2.9, compared to a pH of 9.2. The effect of the pH on the convergence of heavy metals plays a crucial role in the migration and transformation of metals. For instance, Yang et al. [48] found that the speciation of Cd in sediment increases with the increase in pH from 4.5–9.5. Zhang et al. [49] found the release tendency of Ni and Cu to be lower in a pH range of 5–10, compared to pH 0–4, probably because of the low concentration of dissolved organic carbon (DOC) in deionized water.

**Figure 16:**
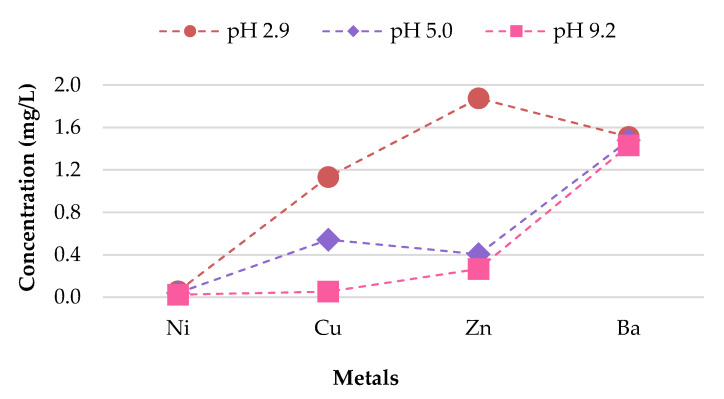
Variable relationship observed between metal concentrations leached from 1% CB bricks and pH value.

**Figure 17:**
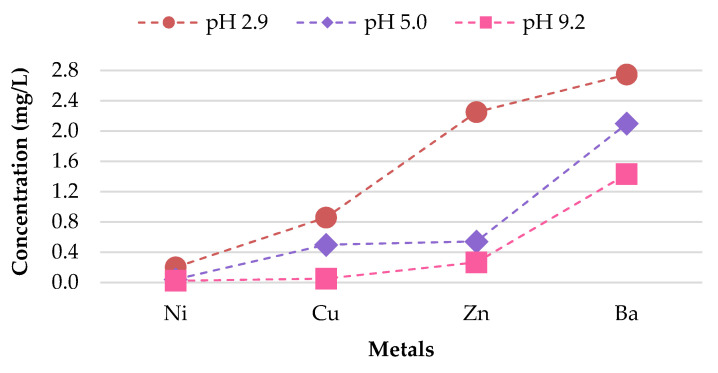
Variable relationship observed between metal concentrations leached from 2% CB bricks and pH value.

A direct relationship between the pH and the metal concentration leachate was found. A decrease in pH from 9.2–2.9 resulted in an increase in the metal concentration leached for Zn, Cu, Ba, and Ni for bricks incorporating 1% and 2% CBs by mass (Figure 16 and Figure 17). This implies that pH values, such as those tested in areas with highly acidic rain (pH 2.9) outside the natural scope of precipitation, will result in the metal contamination of Zn, Cu, Ba, and Ni. This is because acidic water comprises a high number of H^+^ ions. Therefore the surface of the particles is positively charged, which results in weak metal sorption and the release of toxic ions such as Zn, Cu, Ba, and Ni [49]. Other metals, including As, Cr, Se, Ag, Cd, Hg, and Pb are of insignificant concern as the leachate concentrations are below 0.03 mg/L. 

It can be deduced from Table 3 that the metal concentrations of the leachate are trivial when compared with the regulatory threshold limits set by the Industrial Waste Resource Guidelines [34]. The results confirm that fired clay bricks incorporating 0%, 0.5%, 1%, 1.5%, and 2% CBs by mass can be categorized as non-hazardous waste under various pH values as they are clearly below the regulatory limits for heavy metals. To better interpret the insignificance of the results, the leachate concentrations were compared to the United States national primary drinking water thresholds set by the USEPA [35]. Cu and Ba leachate concentrations for 0%, 0.5%, 1%, 1.5%, and 2% CB bricks were the only metals moderately above the threshold limits, while the remaining leachate concentrations were considerably below the limits.

### 3.5. Unfired Bricks and Fired Bricks Comparative Leachate Analysis

**Figure 18:**
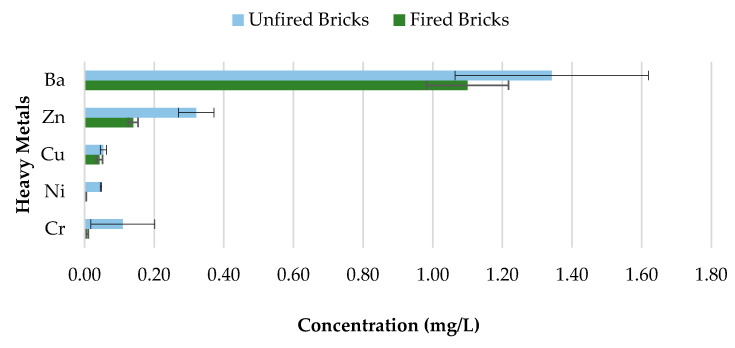
Heavy metal concentration of 0.5% CB content bricks before and after firing.

**Figure 19:**
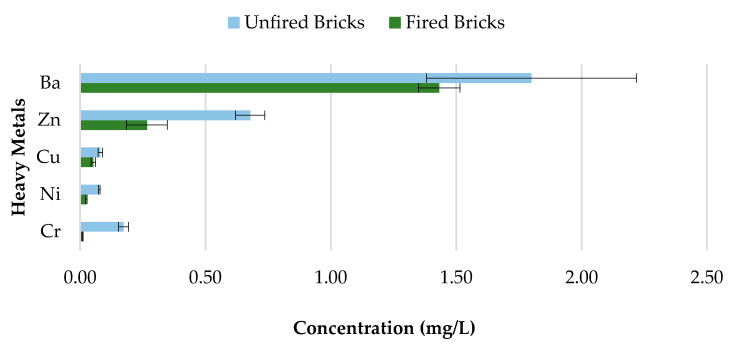
Heavy metal concentration of 1% CB content bricks before and after firing.

**Figure 20:**
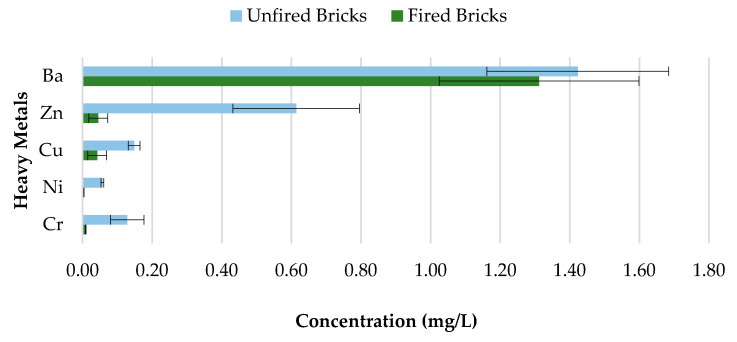
Heavy metal concentration of 1.5% CB content bricks before and after firing.

**Figure 21:**
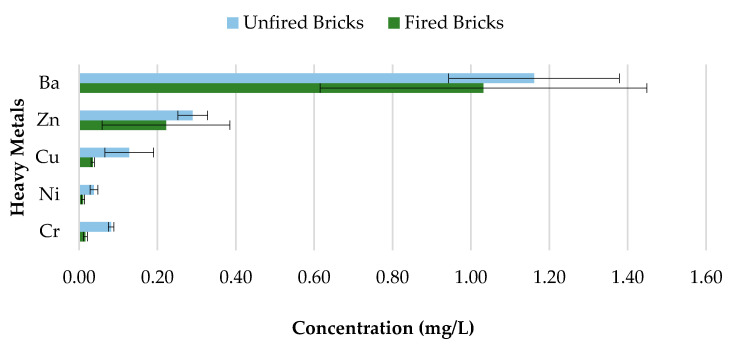
Heavy metal concentration of 2% CB content bricks before and after firing.

Concerns about heavy metal contamination to the environment that might be initiated through the incorporation of CBs, which cannot be combusted during the firing process, represent a major issue. Therefore, to investigate the variations in concentrations of 0%, 0.5%, 1%, 1.5%, and 2% CB bricks before and after firing, pH value 9.2 was selected. The results are presented in Figure 18, Figure 19, Figure 20 and Figure 21 and Table 4. All data are means of three values relative to standard deviation. The findings show that the leaching behavior of the fired clay bricks is notably lower after the firing process. This is the result of the suppression of metals that are locked in the bricks when fired to high temperatures. This behavior can be associated with the silicate-based physio chemical containment process of fired clay bricks [50]. The main compounds found in the brick soil were silica (SiO_2_) and iron oxide (Fe_2_O_3_). Silica enhances the interconnection and compactness of the material, which can result in metal immobilization [51], while iron oxide can assist in the formation and stability of the material, because of the crystallization-dehydration of less crystallized free iron oxides and the removal of organic materials [52], hence decreasing the leaching potential of heavy metals from the fired clay bricks incorporating CBs.

The fluctuations in the leachate concentrations for unfired and fired bricks incorporating 0.5%, 1%, 1.5%, and 2% CBs by mass are summarized in Table 4. The results indicate that the addition of CBs in fired clay bricks considerably reduces the leaching ability of heavy metals. For example, the leaching of Cr and Ni was almost completely impeded after firing for bricks incorporating 0.5% and 1.5% CBs by mass. In addition, over 50% of all tested heavy metals were immobilized after firing for 0.5%, 1%, 1.5%, and 2% CB bricks. However, Ba demonstrated the least change in metal contamination after the firing process. More than 90% of Cr was immobilized in CB fired clay bricks compared to CB unfired bricks. Incorporating CBs in fired clay bricks resulted in a decrease in the metal contamination of the toxic waste, which can be an environmental advantage compared to the littering of CBs. The high firing temperature immobilized the heavy metals within the brick due to the crystalline structure of the material. 

To further study the leaching performance of toxic metals at various liquid to solid ratios and for compacted granular material, leaching tests can be conducted according to Method 1314 [30] and Method 1315 [31]. Although the leaching procedure followed in this study considers a wide range of pH values subject to changing environmental conditions, it does not consider leaching subject to various liquid to solid ratios and compacted granular material. However, it can be stated that fired clay bricks amended with CBs are safe under various conditions and environments as the results are significantly below the threshold limits.

## 4. Conclusions

The incorporation of CBs into fired clay bricks could provide a viable resolution to an escalating waste pollution tragedy, with acceptable mechanical and physical properties and a new cost-effective construction product. Therefore, the results presented here may clarify issues raised regarding the possible leachate of heavy metals from the use and disposal of bricks incorporating CBs. The following conclusion can be drawn from this study:

- Various leachate testing methods were compared, including the ABLP and USEPA methods. In reviewing the various leaching test procedures, the ABLP was determined to be the most suitable leaching procedure for this study based on the sample size, pH values, disposal-to-land scenarios, and condition of the sample.
- Metals Cu, Zn, Mn, Al, Fe, Ti, and Ba demonstrated the highest leachate concentration for pH 2.9 and 5.0 for used CBs. This implies that used CBs are more likely to leach heavy metals in areas with highly acidic rain compared to the natural range of precipitation. This is due to the enhancement of metal mobilization in acidic water, whereby the toxic ions (toxic metals) bound to the negatively charged surface and are displaced by H^+^ (hydrogen) ion.
- It was found that used and shredded used CBs are more prone to leaching heavy metals Cr, Cd, Zn, Mn, Al, and Fe compared to unused CBs. This is because an enhanced level of Cr, Cd, Zn, Mn, Al, and Fe is absorbed and retained by the filter during combustion.
- The leachate results for fired clay bricks incorporating 0%, 0.5%, 1%, 1.5%, and 2% CBs by mass were found to be below the regulatory limits set by the EPA Victoria solid industrial waste guidelines. Therefore, bricks modified with CBs can be categorized as non-hazardous waste.
- The leaching of Cr and Ni was almost completely hindered after the firing process of clay bricks incorporating 0.5% and 1.5% CBs by mass. In addition, more than 50% of all the tested heavy metals were immobilized after firing for 0.5%, 1%, 1.5%, and 2% CBs by mass bricks. Hence the addition of CBs in fired clay bricks may be a feasible option to hinder the heavy metals present in CBs.
- The compressive strength of the CB brick samples varied considerably with the percentage of organic content within the mixture. However, the values were considerably above the minimum requirement of 17.2 MPa for compressive strength. The addition of CBs produced less dense and porous bricks, with better thermal insulation properties.
- The water absorption and IRA results were within the acceptable range, indicating CB brick samples are competent to endure harsh weather conditions.

It is recommended to investigate the leaching performance of toxic metals at the various liquid-to-solid ratios and for compacted granular material, as described in Method 1314 [30] and Method 1315 [31], to consider leaching of bricks subjected to various disposal or use scenarios. Moreover, acceptable limits for heavy metals for various applications, particularly for construction material manufactured with waste material, should be developed. However, it can be stated that fired clay bricks incorporating CBs are safe under various conditions and environments as the leachate concentrations are adequately below the regulatory limits. Moreover, the addition of CB waste in brick manufacturing offers a sustainable recycling solution to a growing pollution issue.

## Figures and Tables

**Table 1:** Applications and limitations of various leachate testing methods.

Method	Applications	Limitations	References
Australian Bottle Leaching Procedure (ABLP) (AS 4439.3)	Inorganic and semi-volatile organic constituents Range of disposal-to-land scenarios Applicable for liquid and solid wastes	Only suitable for particle sizes less than 2.4 mm Not applicable for encapsulated wastes	[26]
Toxicity Characteristics Leaching Procedure (TCLP) (Method 1311)	Aerobic conditions Organic and inorganic analytes Applicable for liquid, solid and multiphasic wastes	Not suitable for anaerobic conditions Suitable only for short-term leaching	[27]
Synthetic Precipitations Leaching Procedure (SPLP) (Method 1312)	Inorganic and organic constituents Volatile organic material leachate Aerobic conditions	Suitable only for aerobic conditions Suitable only for short-term leaching Only applicable for particle size samples	[28]
Liquid-Solid Partitioning as a Function of Extract pH using a Parallel Batch Extraction Procedure (Method 1313)	Inorganic and semi-volatile organic constituents Non-volatile organic constituents Evaluation of disposal, beneficial use, treatment effectiveness and site remedies Suitable for a wide range of solid materials and pH values	Not applicable for characterizing the release of volatile organic analytes	[29]
Liquid-Solid Partitioning as a Function of Liquid-Solid Ratio for Constituents in Solid Materials Using an Up-Flow Percolation Column Procedure (Method 1314)	Inorganic and non-volatile organic constituents Evaluation of disposal, beneficial use, treatment effectiveness and site remedies Suitable for a wide range of granular materials Provides options for the preparation of analytical samples	Not suitable for monolithic materials without particle size reduction Not applicable for characterizing the release of volatile organic analytes	[30]
Mass Transfer Rates of Constituents in Monolithic or Compacted Granular Materials Using a Semi-dynamic Tank Leaching Procedure (Method 1315)	Inorganic analytes Monolithic or compacted granular material Suitable for a wide range of solid materials	Not applicable for the release of organic analytes Does not provide a solution considered under field conditions Minimum sample size 5 cm	[31]
Method 1316: Liquid-Solid Partitioning as a Function of Liquid-to-Solid Ratio in Solid Materials Using a Parallel Batch Procedure (Method 1316)	Inorganic and non-volatile organic constituents Natural pH of the solid material Suitable for a wide range of solid materials	Not applicable to characterize the release of volatile organic analytes Does not provide a solution considered under field conditions	[32]
Characterization of Waste—Leaching—Compliance Test for Leaching of Granular Waste Materials and Sludges—Part 1	Inorganic constituents Monolithic or granular material	Not applicable for non-polar organic constituents Only suitable for particle sizes less than 4 mm	[33]

**Table 2:** Properties of brick samples with 0%, 0.5%, 1%, 1.5%, and 2% CB content.

Mixture Identification (%)	Height Shrinkage (%)	Diametric Shrinkage (%)	Average Density (kg m^−3^)	Compressive Strength (MPa)	Average Water Absorption (%)	Initial Rate of Absorption (kg m^−2^ min^−1^)
CB (0)	3.07	4.64	2114.33	48.64	9.0	0.44
CB (0.5)	2.27	3.89	2025.5	34.31	10.3	0.58
CB (1.0)	2.17	3.45	1983	30.78	12.1	0.67
CB (1.5)	2.1	3.41	1971.8	28.34	12.9	0.75
CB (2.0)	1.96	3.1	1969.22	27.2	13.1	0.84

**Table 3:** Australian Bottle Leaching Procedure (ABLP) (<2.4 mm) leachate results for heavy metals in crushed brick samples using inductively coupled plasma mass spectrometry (ICP-MS).

Heavy Metals	Concentration Level (mg/L)		Percentages of CBs by Mass (mg/L)														
			0%			0.5%			1%			1.5%			2%		
	USEPA Drinking Water ^a^	EPA Vic Solid Waste ^b^	pH 2.9	pH 5.0	pH 9.2	pH 2.9	pH 5.0	pH 9.2	pH 2.9	pH 5.0	pH 9.2	pH 2.9	pH 5.0	pH 9.2	pH 2.9	pH 5.0	pH 9.2
**Cr**	0.1	2.5	0.004 ± 0.004	0.014 ± 0.003	0.009 ± 0.003	0.005 ± 0.001	0.015 ± 0.003	0.007 ± 0.004	0.008 ± 0.001	0.013 ± 0.003	0.010 ± 0.002	0.007 ± 0.001	0.012 ± 0.003	0.009 ± 0.001	0.011 ± 0.002	0.016 ± 0.001	0.016 ± 0.005
**Ni**	-	1	0.007 ± 0.006	0.044 ± 0.004	0.004 ± 0.004	0.010 ± 0.005	0.043 ± 0.012	0.003 ± 0.002	0.056 ± 0.005	0.041 ± 0.013	0.025 ± 0.003	0.008 ± 0.004	0.039 ± 0.013	0.003 ± 0.001	0.203 ± 0.163	0.043 ± 0.015	0.010 ± 0.004
**Cu**	1.3	100	1.559 ± 0.331	0.523 ± 0.128	0.035 ± 0.009	1.536 ± 0.295	0.557 ± 0.082	0.043 ± 0.009	1.134 ± 0.472	0.543 ± 0.099	0.053 ± 0.009	1.496 ± 0.136	0.480 ± 0.208	0.042 ± 0.027	0.860 ± 0.076	0.499 ± 0.122	0.035 ± 0.004
**Zn**	-	150	1.792 ± 0.181	0.628 ± 0.016	0.104 ± 0.092	1.909 ± 0.174	0.645 ± 0.195	0.140 ± 0.014	1.876 ± 0.291	0.406 ± 0.058	0.267 ± 0.081	1.867 ± 0.337	0.335 ± 0.095	0.045 ± 0.027	2.251 ± 0.487	0.543 ± 0.076	0.222 ± 0.163
**As**	0.01	0.35	0.005 ± 0.001	0.010 ± 0.009	0.003 ± 0.001	0.011 ± 0.002	0.011 ± 0.006	0.005 ± 0.001	0.004 ± 0.003	0.014 ± 0.001	0.006 ± 0.002	0.044 ± 0.012	0.038 ± 0.025	0.015 ± 0.002	0.005 ± 0.001	0.015 ± 0.003	0.001 ± 0.000
**Se**	0.05	0.5	-	0.002 ± 0.001	-	0.002 ± 0.001	0.002 ± 0.001	-	0.013 ± 0.001	0.002 ± 0.001	0.003 ± 0.002	0.003 ± 0.001	0.003 ± 0.001	0.002 ± 0.001	0.015 ± 0.001	0.003 ± 0.001	-
**Ag**	-	5	-	-	-	-	0.009 ± 0.004	-	-	-	-	-	-	-	-	0.001 ± 0.000	-
**Cd**	0.005	0.1	0.001 ± 0.001	-	-	-	0.001 ± 0.000	-	-	0.001 ± 0.000	-	-	-	-	-	0.001 ± 0.001	-
**Ba**	2	35	1.450 ± 0.168	1.648 ± 0.138	1.447 ± 0.435	1.473 ± 0.221	1.770 ± 0.182	1.100 ± 0.118	1.513 ± 0.268	1.481 ± 0.075	1.432 ± 0.083	1.530 ± 0.14	1.526 ± 0.222	1.312 ± 0.287	2.747 ± 0.984	2.098 ± 0.566	1.032 ± 0.417
**Hg**	0.002	0.05	-	-	-	-	-	-	-	-	-	-	-	-	-	-	-
**Pb**	0.015	0.5	0.012 ± 0.003	0.010 ± 0.007	0.001 ± 0.001	0.005 ± 0.001	0.008 ± 0.005	0.001 ± 0.000	0.050 ± 0.028	0.006 ± 0.002	0.006 ± 0.001	0.003 ± 0.003	0.007 ± 0.001	0.001 ± 0.001	0.011 ± 0.004	0.010 ± 0.008	0.002 ± 0.001

^a^ USEPA [35]. ^b^ Industrial Waste Resource Guidelines [34]. Note: All data are means of three values.

**Table 4:** Leaching concentrations of unfired and fired bricks containing 0.5%, 0.5%, 1%, 1.5%, and 2% CBs by mass for pH 9.2.

Heavy Metals	Percentage of Leaching Drop before and after Firing (%)											
	0.5%			1%			1.5%			2%		
	Unfired Bricks	Fired Bricks	% Leaching Drop	Unfired Bricks	Fired Bricks	% Leaching Drop	Unfired Bricks	Fired Bricks	% Leaching Drop	Unfired Bricks	Fired Bricks	% Leaching Drop
**Cr**	0.110 ± 0.092	0.007 ± 0.004	94	0.174 ± 0.020	0.010 ± 0.002	94	0.128 ± 0.048	0.009 ± 0.001	93	0.082 ± 0.007	0.016 ± 0.005	80
**Ni**	0.047 ± 0.002	0.003 ± 0.002	94	0.077 ± 0.003	0.025 ± 0.003	68	0.057 ± 0.004	0.003 ± 0.001	95	0.038 ± 0.010	0.010 ± 0.004	74
**Cu**	0.054 ± 0.009	0.043 ± 0.009	20	0.081 ± 0.009	0.053 ± 0.009	35	0.148 ± 0.017	0.042 ± 0.027	72	0.128 ± 0.062	0.035 ± 0.004	73
**Zn**	0.321 ± 0.051	0.140 ± 0.014	56	0.678 ± 0.058	0.267 ± 0.081	61	0.614 ± 0.182	0.045 ± 0.027	93	0.290 ± 0.038	0.222 ± 0.163	23
**Ba**	1.342 ± 0.278	1.100 ± 0.118	18	1.800 ± 0.419	1.432 ± 0.083	20	1.423 ± 0.261	1.312 ± 0.287	8	1.161 ± 0.218	1.032 ± 0.417	11

Note: All data are means of three values.
